# Development of an indirect ELISA for detecting humoral immunodominant proteins of *Mycoplasma hyopneumoniae* which can discriminate between inactivated bacterin-induced hyperimmune sera and convalescent sera

**DOI:** 10.1186/s12917-019-2077-4

**Published:** 2019-09-12

**Authors:** Honglei Ding, Yaoqin Zhou, Haoju Wang

**Affiliations:** Laboratory of Veterinary Lemology, College of Animal Science and Technology, Southwest University, No. 2, Tiansheng Road, Beibei District, Chongqing Municipality, 400715 China

**Keywords:** *Mycoplasma hyopneumoniae*, Indirect ELISA, Immunodominant protein, Hyperimmune sera, Convalescent sera

## Abstract

**Background:**

*Mycoplasma hyopneumoniae* (*M. hyopneumoniae*) is the primary pathogen of porcine enzootic pneumonia, which has been associated with economic losses due to reduced daily weight gain and feed efficiency. Although it has a small genome and no more than 1000 genes, *M. hyopneumoniae* can be cultured in cell free media. However, some proteins were not expressed or were only expressed in negligible amounts under culture conditions. Nevertheless, some of these proteins can be expressed at a high level and induce a strong and rapid immune response after *M. hyopneumoniae* infection. The unexpressed or less expressed proteins may play critical roles in pathogenesis and/or immune response. In order to find the differentially expressed proteins of *M. hyopneumoniae* between culture condition and infected animals, we established an indirect ELISA for the detection of humoral immunodominant proteins which can discriminate between inactivated bacterin-induced hyperimmune sera and convalescent sera by using Mhp366 protein which did not react with sera from bacterin-immunized pigs, but revealed a strong immunoreaction with porcine convalescent sera.

**Results:**

The checkerboard titration method was done by using porcine convalescent sera as positive sera and inactivated bacterin-induced hyperimmune sera as negative sera. The bacterial lysates of fusion proteins and free GST protein without dilution were the optimal coating antigens. The optimal blocking buffer was PBS with 10% FBS and 2.5% skimmed milk. In the checkboard ELISAs, when the sera were diluted at 1:500 and the HRP-labeled rabbit anti-pig IgG were diluted at 1:20000, most positive result was obtained for the assay.

**Conclusions:**

This established indirect ELISA can be used as a tool for the detection of humoral immunodominant proteins of *M. hyopneumoniae* which can discriminate between inactivated bacterin-induced hyperimmune sera and convalescent sera.

## Background

*Mycoplasma hyopneumoniae* (*M. hyopneumoniae*) is a ubiquitous, fastidious and economically significant pathogen of pig that causes porcine enzootic pneumonia (PEP), resulting in reduced daily weight gain and feed conversion efficiency and predisposed pigs to secondary pathogens, such as *Pasteurella multocida*, *Actinobacillus pleuropneumoniae*, porcine reproductive and respiratory syndrome virus and H1N1 swine influenza virus [1]. Vaccination is the most widely used strategy worldwide to control PEP, combined with hygiene and management procedures and antimicrobial application [2–4]. Commercial vaccines mostly consist of inactivated, adjuvanted whole-cell preparations that are administered intramuscularly [4], although a live *M. hyopneumoniae* vaccine was also applied limitedly by intrapulmonary immunization in China [5, 6]. Inactivated vaccines reduce clinical signs and lung lesions, and improve productive performance, although not significantly [7]. Meanwhile, inactivated vaccines reduce the number of pathogens in the respiratory tract [8]. However, some studies indicate that vaccination does not significantly reduce the transmission of this respiratory pathogen in vaccinated herds compared to unvaccinated ones [7–9].

Recent studies indicated that some proteins were not expressed or only expressed in negligible amounts under culture conditions [10–12]. Nevertheless, some of these proteins can be expressed at a high level and induce a strong and rapid immune response after *M. hyopneumoniae* infection [10]. It hypothesized that the unexpressed or less expressed proteins might play critical roles in protective immunity. Finding the differentially expressed proteins of *M. hyopneumoniae* between culture condition and infected animals can provide candidate antigens for new vaccine investigation, especially recombinant subunit vaccine.

Porcine convalescent serum revealed a strong immunoreaction to Mhp366 protein which did not react with sera from bacterin-immunized pigs. Moreover, Mhp366 in total cell lysates of *M. hyopneumoniae* strains cultured in cell free liquid medium was not detected by using a polyclonal serum raised against Mhp366 [10].

In this study, we use Mhp366 as the antigen to establish an indirect ELISA for the detection of humoral immunodominant proteins which can discriminate between inactivated bacterin-induced hyperimmune and convalescent sera. Meanwhile, we optimize the reactive condition and parameter for further detection of more proteins only expressed sufficiently to stimulate immune response in infected animals.

## Results

### Classification of sera by ELISA and detection of *M. hyopneumoniae* in BALF by nested PCR

All sera were checked by indirect ELISA kit (Table 1). Samples collected from Farm A were all positive for *M. hyopneumoniae* after vaccinating against commercial *M. hyopneumoniae* inactivated vaccine for 4 weeks. All sera were positive in Farm B. However, 8 sera were judged as positive and 12 were negative in Farm C. *M. hyopneumoniae* in BALF samples were detected by nested PCR (Table 1). Compared to serological result, no *P36* gene was detected in Farm A. In farm B, 40% pigs were negative for *M. hyopneumoniae*. However, prevalence of *M. hyopneumoniae* in Farm C (15/20, 75%) was more. Finally, 9 sera in Farm A and 15 positive sera which were coincided with *M. hyopneumoniae* infection in the same pigs in Farm B and Farm C were picked randomly for the following assay. Eight sera were from Farm B and 7 sera were from Farm C.

**Table 1:** Prevalence of *M. hyopneumoniae* infection and *M. hyopneumoniae* positive sera in selected pigs from 3 farms

Farm	No. of pigs	PCR result of BALFs		Commercial ELISA results of sera	
		+	–	+	–
A	20	0	20	20	0
B	20	12	8	20	0
C	20	15	5	8	12

### Expression and purification of Mhp366 protein

**Fig. 1:**
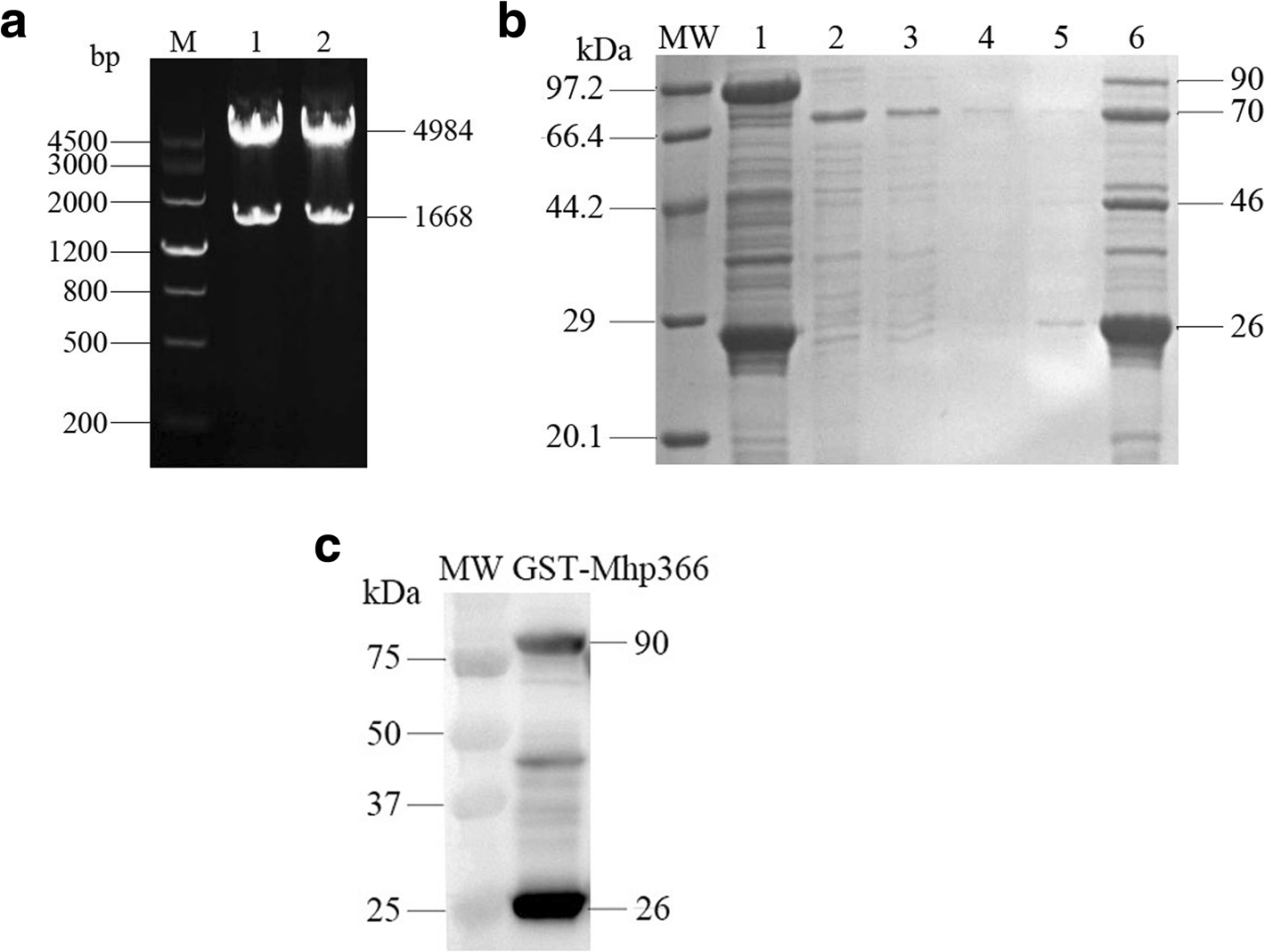
Expression, purification, and identification of Mhp366 protein. **a** Identification of recombinant plasmid pGEX-6P-2-mhp366 by double restriction digestion. Recombinant plasmid pGEX-6P-2-mhp366 was digested with *Bam*HI and *Xho*I and cleaved into pGEX-6P-2 and *mhp366* gene (lane 1, 2). M, DNA marker. **b** Purification of GST-Mhp366 and cleavage of Mhp366 protein off from GST-Mhp366 by PreScission Protease. Mhp366 was cleaved off from the agarose bead-immobilized GST-Mhp366 fusion protein (lane 1) using PreScission protease. A precision protease site is encoded by the pGEX-6P-1 expression vector between GST and Mhp366. After the cleavage, the supernatant was inhaled (lane 2) and the beads were washed three times sequentially (lanes 3, 4, and 5). After digestion and washing, the remaining bead sample was loaded in lane 6. The 90 kDa bands in lane 1 and 6 were GST-Mhp366, 70 kDa bands in lane 2, 3, 4, 5 and 6 were Mhp366, 46 kDa band in lane 6 was PreScission protease, and 26 kDa bands in lane 1 and 6 were GST. MW: protein molecular weight. **c** Western blotting analysis of GST-Mhp366 expression. The GST-Mhp366 was probed with GST-Tag monoclonal antibody. It is worth noting that GST-Mhp366 and GST proteins visualized with Coomassie blue dye in (**b**) were expressed veritably. The 90 kDa band was GST-Mhp366, 26 kDa band was GST, the band about 48 kDa was a nonspecific protein

The recombinant plasmid was verified by nucleotide sequencing and enzyme digestion with *Bam*HI and *Xho*I (Fig. 1a). The recombinant plasmid was transformed into *E. coli* BL21(DE3). IPTG-induced bacteria overexpressed a GST fusion protein in soluble form. The size of the fusion protein was observed on an SDS-polyacrylamide gel. An approximately 90 kDa protein was obtained from the bacterial lysate with purification by using glutathione-conjugated agarose beads. Mhp366 protein about 70 kDa was purified by cleaving off the Mhp366 portion with PreScission protease from the GST-Mhp366 fusion protein immobilized onto the glutathione-agarose beads (Fig. 1b). Fusion protein of GST-Mhp366 was confirmed by western blotting using GST-Tag monoclonal antibody that showed immunoreactivity with approximately 90 kDa recombinant protein (Fig. 1c).

### Optimization of ELISA procedure

**Fig. 2:**
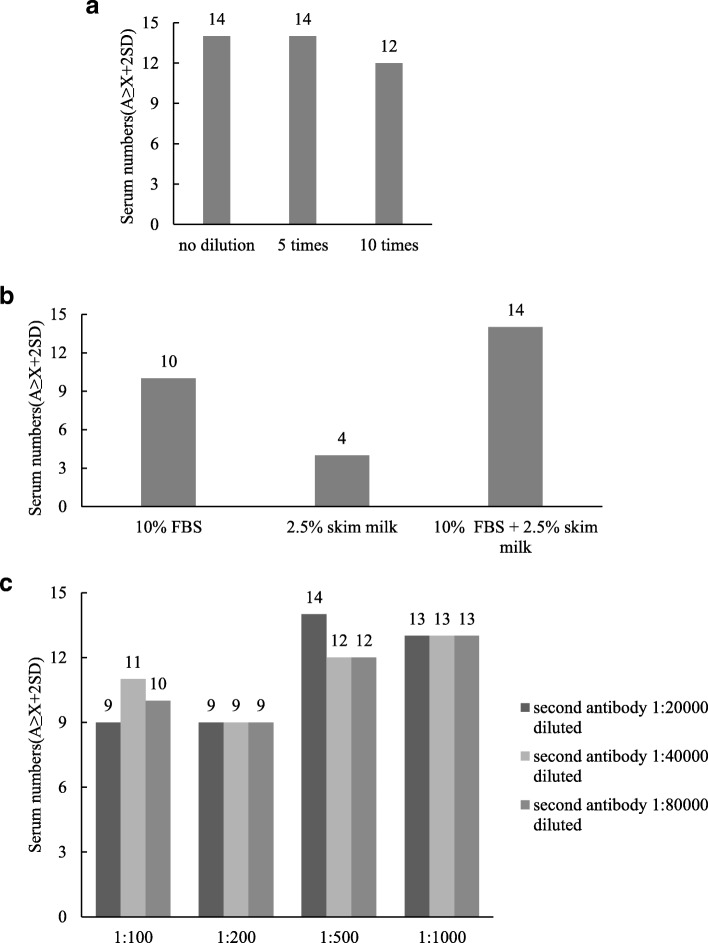
Optimization of ELISA working condition. **a** The optimal concentration of antigen was the bacterial lysates of fusion protein and free GST protein without dilution. **b** The optimal blocking buffer was PBS comprising 10% FBS and 2.5% skimmed milk. **c** Both hyperimmune serum and convalescent serum with dilution of 1:500 and HRP-labeled rabbit anti-pig IgG diluted at 1:20000 were the working concentration of primary antibodies and secondary antibody

Without dilution or at 1:5 dilution, the bacterial lysates of GST-Mhp366 fusion protein and free GST protein were coated with glutathione-conjugated wells, more positive convalescent sera (14 positive convalescent sera) were found, compared to 1:10 dilution (12 positive convalescent sera). Considering the amount of fusion proteins in other recombinant bacteria constructed in the future may be less than Mhp366 recombinant bacterium, in order to provide sufficient fusion protein for saturating the assay plate, bacterial lysate of fusion protein without dilution was considered as the optimal antigen concentration (Fig. 2a).

The bacterial lysates of GST-Mhp366 fusion protein and free GST protein without dilution were added to glutathione-conjugated microplates without dilution. The plates blocked with PBS + 10% FBS + 2.5% skimmed milk gave most positive convalescent sera and the number was 14. However, blocking buffer consisting of 10% FBS or 2.5% skimmed milk gave 10 and 4 positive sera, respectively (Fig. 2b).

In the checkboard ELISAs, when the sera were diluted at 1:500 and the HRP-labeled rabbit anti-pig IgG were diluted at 1:20000, most A ≥ X *+* 2SD result (14 positive convalescent sera) was obtained. Therefore, the optimal working concentration of serum and second antibody were at dilutions of 1:500 and 1:20000, respectively (Fig. 2).

## Discussion

Vaccination with whole-cell inactivated commercial bacterins is commonly applied in swine producing areas to control *M. hyopneumoniae* infections [13]. In many countries, vaccination for controlling *M. hyopneumoniae* infection is applied in more than 70% of the pig herds [1]. In the United States, the application is above 65% [14]. Despite the fact that vaccination against *M. hyopneumoniae* is widely applied, many aspects about its function on prevention of the organism transmission, effect on different strains and on the immunological response remain unclear and need to be further explored. Some studies indicated that bacterins mainly induced humoral immune response [15, 16]. The correlation between the induction of specific antibodies and protection against pneumonia is unclear. Challenge experiments in pigs have shown that the concentrations of antibodies in sera were not correlated with protection against *M. hyopneumoniae* [17]. Thus, the presence and concentration of antibodies in sera does not appear to be the best way to evaluate protective immunity.

Proteomic analysis carried out by Pendarvis et al. indicated 483 of 691 (70%) proteins in *M. hyopneumoniae* 232 strain were expressed under the culture conditions [11]. Tacchi et al. identified 52% (347/666) of predicted proteins by analyzing the proteome of *M. hyopneumoniae*-type strain J grown in modified Friis broth [18]. Partial protein expression of *M. hyopneumoniae* under cell free condition was confirmed by some immunological assays [19, 20]. For example, the membrane proteins MHP0513 and MHP0612 which are not expressed in cell free culturing *M. hyopneumoniae* were recognized by convalescent sera but not hyperimmune sera [19]. Hypothetical protein MHP0596, which could not be detected under in vitro culture, induced antibody in *M. hyopneumoniae* infected pigs [20]. How about the role of the antibodies, which were induced in *M. hyopneumoniae* infected pigs but not stimulated by inactivated vaccine, in the protective immunity? If these antibodies can play important roles in protective immunity, the poor immune protective effects of inactivated vaccine will be interpreted partially.

Development of a method for the detection of humoral immunodominant proteins which can discriminate between inactivated bacterin-induced hyperimmune sera and convalescent sera can provide a powerful and useful tool for evaluating the proteins which raise antibodies only during the time when *M. hyopneumoniae* infects pigs. Meens et al. indicate porcine convalescent sera induced a strong immunoreaction on the Mhp366 protein which did not react with sera from bacterin-immunized pigs [10]. In addition, it was not possible to detect Mhp366 in total cell lysate of in vitro grown *M. hyopneumoniae* strains, using a polyclonal rabbit serum raised against Mhp366 protein. Therefore, we established an indirect ELISA which can discriminate between inactivated bacterin-induced hyperimmune sera and convalescent sera based on Mhp366 protein.

*Mycoplasma* sp. use a specific genetic code. The amino acid tryptophan is not encoded by TGG, but by TGA, which is a stop codon in most organisms [21, 22]. This difference has retarded the expression of genes of *M. hyopneumoniae* containing TGA stop codons in *E. coli*, the most seductive system used for production of recombinant proteins [23]. However, mutations that can replace TGA codons with TGG have been widely used to deal with this problem. However, site-directed mutation is time-consuming; especially there are more TGA codons in one gene. In order to reduce workload of the operators, we synthesized the *mhp366* gene and mutate the TGA codon encoded tryptophan to TGG while synthesizing the gene. This procedure shortened the hours and labors enormously.

In theory, most of the proteins with immune protection are membrane proteins. However, it is often difficult to express membrane proteins in soluble form using prokaryotic expression system. They are usually expressed as insoluble particles named inclusion body. The insoluble particles should be denatured and refolded by a complex process. Generally, a pGEX vector system allows fusion protein to be expressed in soluble form, for the GST fusion tag is an extreme hydrophilic protein. Therefore, this vector system will reduce the hours and labors for protein renaturation.

Pig sera may contain antibodies that are reactive with bacterial antigens that potentially contaminate the microplate wells during the fusion protein array. To minimize the detection of cross-reactive antibodies, all serum samples were preabsorbed with bacterial lysate of free GST induced with IPTG. In order to minimize the background, we used OD630 from bacterial lysate of GST-Mhp366 to minus OD630 from bacterial lysate of free GST. This procedure reduced the trouble for protein purification. It is usually difficult to decide the cut-off value in the standardization of an ELISA, since a continuous change such as OD must be converted into a qualitative response (positive or negative) [24]. Many criteria have been applied to the cut-off value of ELISA previously, such as the mean of the sample to positive ratio value plus 2 standard deviations of the control sera. Another cut-off value was judged by using a Receiver Operating Characteristic (ROC) curve analysis [25, 26], which leads to the high sensitivity and specificity of the ELISA [27] and the cut-off value was calculated as the average value obtained from the negative control serum samples plus 3 standard deviations. The selected cut-off values in these studies used the difference of mean OD630 of hyperimmune sera reacted with bacterial lysates of GST-Mhp366 and free GST (negative) plus 2 standard deviations due to the convenience of calculation and availability in practice.

## Conclusion

In summary, an indirect ELISA method has been effectively developed. The method used bacterial lysates of fusion protein and free GST protein without dilution as the coating antigens and blocked with PBS adding 10% FBS and 2.5% skimmed milk. The optimal conditions of pig sera dilution and HRP-conjugated rabbit anti-pig IgG antibody dilution were 1:500 and 1:20000, respectively. The cut-off value was the difference of mean OD630 of hyperimmune sera (bacterial lysates of fusion protein minus free GST protein) plus 2 standard deviations. This suggests that this indirect ELISA can be used as a tool for the detection of humoral immunodominant proteins of *M. hyopneumoniae* which can discriminate between inactivated bacterin-induced hyperimmune sera and convalescent sera.

## Methods

### Animal source

Serum samples used in this study were collected from 3 farms. Pigs in Farm A were *M. hyopneumoniae*-free and no PEP-like clinical symptoms or lung lesions were observed. Pathogen and serology detection were carried out in recent 2 years. *M. hyopneumoniae* organism detection was performed by nested-PCR [25] and culturing in KM2 cell-free liquid medium [28] in the presence of 2 μg/mL kanamycin [29] and 100 μg/mL ampicillin. Serological detection of *hyopneumoniae* IgG antibody was performed with a commercial ELISA kit (IDEXX Inc., USA). *M. hyopneumoniae* organisms and nucleotide are free by culture and nested PCR. Also, the sera are negative by immunological diagnosis with commercial ELISA kit. Pigs in Farm B and C with a history of PEP according to clinical observation and serological surveillance in recent 2 years. In farm B, PEP sporadically occurred. However, about a third of pigs showed PEP-like clinical syndromes in Farm C. All pigs in farms A, B and C were not immunized with any *M. hyopneumoniae* vaccine.

### Sample collection

In order to get sufficient qualified samples for further study, pigs were selected randomly and blindingly 2–4 times of the needed sera. Twenty pigs from Farm A were immunized with a one-shot commercial *M. hyopneumoniae* inactivated vaccine (MYPRAVAC SUIS, Hipra Lab) on day 21 when they were weaned. MYPRAVAC SUIS is a whole-cell, inactivated bacterin based on J strain, with mineral oil and aluminium hydroxide as adjuvanted. Twenty-eight days after immunization, serum was collected from the front cavity vein of each pig. Serum samples from fattening pigs of 120–200 days were also collected from Farm B and Farm C (Twenty samples were collected from each farm). After collection, *M. hyopneumoniae* IgG antibody were detected with a commercial ELISA kit (IDEXX Inc., USA). Glycerol was added and the final concentration was 50%. Then, the sera were kept in aliquots at − 20 °C until use.

Bronchoalveolar lavage fluids (BALF) were collected by fiberoptic bronchoscopy as described by previous reports [30, 31]. The collected BALF were processed, aliquoted, frozen at − 20 °C immediately for *M. hyopneumoniae* detection by nested PCR as described previously [25]. The samples were concentrated by centrifugation at 12000 g for 30 min, the pellets were resuspended in PBS (pH 7.2) and treated as described by Blanchard et al. [32].

All pigs used in this study were released after sample collection.

### Expression and purification of Mhp366 protein

*Mhp366* gene was synthesized with the modification of 4 TGA codons which encode tryptophan at amino acid positions 279, 323, 422 and 483 in *M. hyopneumoniae* genomes to TGGs (Sangon Biotech, China). Meanwhile, *Bam*HI and *Xho*I restriction sites were added to the 5′ and 3′ end of the synthesized gene, respectively. Then, the synthesized gene was ligated into vector pGEX-6P-1 to construct recombinant plasmid. This vector enables the protein to be overexpressed as a fusion protein with glutathione *S*-transferase (GST) that was fused to the N terminus of the Mhp366 protein [33]. Recombinant plasmid was transformed into *E. coli* BL21(DE3) using a heat shock method. Identified colonies were supplemented with a final concentration of 100 μg/ml ampicillin and incubated on rotary shaker at 37 °C in LB media. Overnight culture was inoculated into LB media with a dilution of 1:100. When the mid-log phase reached to an A_600_ between 0.7–0.9, protein expression was induced at 30 °C for 5 h with isopropyl-β-d-thiogalactoside (IPTG) to a final concentration of 1 mM. After protein induction, bacteria were collected via centrifugation. The bacterial pellets were resuspended in a Triton lysis buffer (1% Triton X-100, 75 IU/ml of aprotinin, 1 mM phenylmethylsulfonyl fluoride, 20 μM leupeptin, and 1.6 μM pepstatin in PBS) with the final volume of 1/20 compared to the bacterial fluid and were lysed by sonication on ice. After a high-speed centrifugation at 12000 g to deposit debris, bacterial soluble fraction was processed and stored at − 80 °C. The expression of the fusion protein was evaluated by purifying the fusion protein from a part of the soluble fraction using glutathione-conjugated agarose beads (GE Healthcare). After thoroughly washing, the Mhp366 protein was cleaved off from the beads with PreScission protease. As a negative control, the empty vector pGEX-6P-1 was also transformed into *E. coli* BL21(DE3), and then induced the expression of GST protein with IPTG. The fusion protein and cleaved protein were assessed by 12% sodium dodecyl sulfate-polyacrylamide gels electrophoresis stained with a Coomassie blue dye.

### Western blotting assay

The purified fusion protein was solubilized in sample buffer (50 mM Tris·HCl pH 6.8, 10% glycerol, 0.1% bromophenol blue, 1% 2-mercaptoethanol, 2% SDS) and loaded onto SDS polyacrylamide gels. After electrophoresis, the proteins were transferred to polyvinylidene difluoride membrane (Roche Diagnostics) for 2 h at 100 V using a transblotting apparatus (Bio-Rad, USA). The membrane was blocked overnight at 4 °C in 5% skimmed milk-TBST and was detected by GST-Tag monoclonal antibody (3A10) (Bioworld Technology, China) with a 1:8000 dilution at RT for 1 h. The primary antibody binding was incubated with a 1:20000 dilution of horseradish peroxidase (HRP)-conjugated goat anti-mouse IgG secondary antibody (Proteintech, China) at RT for 1 h and visualized with an enhanced chemiluminescence kit (CWBio, China).

### Optimization of ELISA procedure and working condition

Bacterial lysates of GST-Mhp366 fusion protein or GST protein expressed in pGEX-6P-1 empty vector were added to glutathione-coated 96-well microplates (GE Heathcare, USA) without dilution or at 1:5, 1:10 dilution with a total volume of 200 μl/well. The plates were incubated overnight at 4 °C to facilitate GST-Mhp366 to bind to the glutathione immobilized on the plate. Following five washes in PBS containing 0.05% Tween-20 (PBST), the plates were blocked with 200 μl PBS adding 10% FBS, 2.5% skimmed milk or 10% FBS + 2.5% skimmed milk at RT for 1 h. Following five washes with PBST, 100 μl porcine serum diluted at 1:100, 1:200, 1:500, 1:1000 with blocking solution was added to each well and incubated at RT for 2 h. To minimize the detection of cross-reactive antibodies, all serum samples were preabsorbed with bacterial lysate of free GST. After five washes with PBST, the plates were conjugated with 100 μl HRP-labeled rabbit anti-pig IgG (Invitrogen, USA) diluted at 1:20000, 1:40000, 1:80000 with blocking solution at 37 °C for 1 h. The plates were washed as described above, and a colorimetric reaction was induced by the addition of chromogenic substrate (Keqian, China) at RT for 10 min. Color development was stopped with 50 ml stop solution (Keqian, China), and the optical density at 630 nm was recorded using a universal Microplate Reader xMark™ (Bio-Rad, USA). A total of 15 convalescent sera and 9 hyperimmue sera were used in the ELISA assay. All convalescent sera used in the assay were positive to *M. hyopneumoniae* and the *P36* gene of *M. hyopneumoniae* in BALF to corresponding serum was also detected as positive.

### Calculation of cut-off value

Nine hyperimmune sera were reacted with bacterial lysates of GST-Mhp366 and free GST, respectively. The difference of optical density (OD) at a wavelength of 630 nm (OD630) from bacterial lysates of GST-Mhp366 and free GST was calculated. The average difference was named X, and the standard deviation was named SD. The cut-off value was calculated as the average difference plus 2 standard deviations (X + 2SD). Fifteen convalescent sera were reacted with bacterial lysates of GST-Mhp366 and free GST, respectively. Identically, the difference of OD630 from bacterial lysates of GST-Mhp366 and free GST was also calculated and named A.

For the interpretation, if A ≥ X *+* 2SD, the convalescent serum was classified as positive. The optimal reaction condition was determined as the maximum number of sera that the OD630 were A ≥ X *+* 2SD.

## Data Availability

The dataset analyzed during the current study is available from the corresponding author on reasonable request.

## Abbreviations

BALF: Bronchoalveolar lavage fluids
ELISA: Enzyme-linked immunosorbent assay
GST: Glutathione *S*-transferase
IPTG: Isopropyl-β-d-thiogalactoside
OD: Optical density
PEP: Porcine enzootic pneumonia
SD: Standard deviations
